# Fundamental Structural and Kinetic Principals of High Strength UHMWPE Fibers Production by Gel-Technology

**DOI:** 10.3390/polym14214771

**Published:** 2022-11-07

**Authors:** Elena Ivan’kova, Viktor Egorov, Vyacheslav Marikhin, Liubov Myasnikova, Yuri Boiko, Elena Radovanova

**Affiliations:** 1 Institute of Macromolecular Compounds of Russian Academy of Sciences, V.O., Bol’shoy pr.31, St. Petersburg 199004, Russia; 2 Ioffe Institute of Russian Academy of Sciences, Polytekhnicheskaya 26, St. Petersburg 194021, Russia

**Keywords:** UHMWPE, reactor powders, xerogel, gel-technology, SEM, WAXS, DSC, mechanical properties

## Abstract

One of the main research work in the field of polymeric materials was, is and always will be the improvement of their mechanical properties. Comprehensive structural studies of UHMWPE reactor powder, the features of its dissolution and the formation of a gel-state, as well as UHMWPE films oriented up to various draw ratios, were carried out using scanning electron microscopy, differential scanning calorimetry, and X-ray diffraction. For comparison, decalin and vaseline oil were chosen as solvents. The mechanical properties of oriented UHMWPE films were also studied. In the process of orientation drawing, basing on the developed structural-kinetic principles of strengthening for highly oriented speciments gel-cast from UHMWPE powders, the average values of tensile strength of 4.7 GPa (about 6% of the samples had strength values up to 6.0 GPa) and an Young’s modulus of 170 GPa (about 6% of the samples had Young’s modulus values of 200 GPa). These values are among the highest according to the world scientific literature. A significant increase in the mechanical characteristics of highly oriented UHMWPE films was achieved using experimentally confirmed scientific approaches to revealing the structure-property relationship at each stage of the gel process.

## 1. Introduction

The problem of obtaining high-strength and high-modulus polymers continues to be extremely relevant due to the ever-increasing requirements for the mechanical properties of materials used both in traditional structural areas and in special-purpose areas, including aircraft- and shipbuilding. These issues are becoming especially topical today in the development of effective means of protection, both for the life of people and the preservation of machinery and equipment in extreme conditions.

For a long time, the mechanical characteristics of polymer fibers based on flexible-chain polymers of polyethylene (PE), polypropylene (PP), polyethylene terephthalate (PET), etc., obtained by the most common melt-technology so far, remained at an average level: their strength did not exceed 1 GPa, and initial modules 25–40 GPa, that significantly limited the scope of their practical application, especially in special-purpose products [1,2]. Only in the 1980s in the Netherlands, after commercial production of ultrahigh molecular weight polyethylene (UHMWPE) with a molecular weight of more than 10^6^ g/mol, a fundamentally new method was developed, which later became known as gel-technology [3]. This method made it possible to radically improve the complex of physical and mechanical characteristics of the UHMWPE fibers. In the very first scientific publications, strength values of 3 GPa and a Young’s modulus of about 120 GPa were given. This makes it possible to attribute UHMWPE fibers to the category of ultra-strong and ultra-high-modulus materials.

The solution to the problem of a significant increase in the physical and mechanical characteristics of the UHMWPE films/fibers obtained by the gel-spinning method is possible only on the basis of a deep analysis of the processes occurring at each stage of the films/fiber production and the development of new science-based approaches to their optimization. The theoretical value of the initial modulus is estimated at 260–340 GPa, and the ultimate tensile strength, which according to theory is 0.1 of the elastic moduli of the molecule, is estimated at 15–18 GPa at room temperature [4]. Thus, there is an undoubted reserve for further improvement of the properties of the UHMWPE fibers, although its use is a very difficult task.

This is evidenced by at least the fact that since the establishment of industrial production at DSM and Allied Sign.Inc.-Honeywell in the 80s of the last century, despite the large number of research papers devoted to gel technology [3,5,6], special progress in improving the mechanical properties of manufactured products is not observed.

Obviously, this is due both to the technological difficulties of the most multi-stage process of orientation drawing, and, especially, to an insufficient understanding of the regularities of physical phenomena occurring at each stage of material processing. We believe that the use of fundamental data accumulated in the world scientific literature, as well as data obtained during the present study, will serve as the basis for the future scientifically based modernization of the existing gel-process and will lead to an increase in the complex of physical and mechanical characteristics of the UHMWPE fibers and films.

The gel-technological process for obtaining high-strength and high-modulus fibers is realized in several stages, including the selection of a UHMWPE reactor powder, the preparation of a specific spinning solution at high temperatures, the formation of filaments by forcing the solution through spinnerets, the formation of gel fibers while cooling the filaments, and the final multi-stage orientation drawing. At each stage of the process, there is a problem of choosing certain parameters, the optimization of which should lead to an increase in the physical and mechanical properties of the fibers.

The special advantages of gel-technology over melt-technology are determined by the formation in the initial unoriented state of a specific morphology such as a crystalline mat from fairly perfect lamellar crystals layered on top of each other, the so-called xerogel. The nature of this structure formed in the course of crystallization during cooling of the gel-solution substantially controls its ability to high orientational elongations, which can be 15–20 times higher than the draw ratio of the same polymer crystallized from the melt [4,6]. Therefore, a great attention should be given to the problem of finding the optimal xerogel structure that ensures the achievement of the highest physical and mechanical properties of UHMWPE filaments.

Smith and Lemstra [7,8,9,10] showed in their early work that even low molecular weight PE M_w_ = 2.8 × 10^5^ can be used in gel-technology. However, in order to obtain filers/films with high mechanical properties, significantly higher values of molecular weights are required. It has been experimentally and theoretically shown that, in this case, the degrees of orientational drawing increase significantly and, as a result, the strength and initial modulus evidently rise [9,10,11,12,13]. At present, it is difficult to say what maximum values of molecular weight can be achieved in the process of synthesis of PE. In a number of laboratory studies [14,15,16,17,18,19,20] and experimental technological processes [20,21,22,23,24,25], reactor powders from M_w_ = 3 × 10^6^ to M_w_ = 10^7^ were used. An important factor is the low number of side CH_3_ branches and double bonds in the skeleton of the macromolecule. Some authors have studied the effect of molecular weight distribution on the mechanical properties of oriented fibers [26,27,28].

It is believed that the choice of solvent type is not critical, but this has not been verified experimentally. In [9,20,23,29] a list of numerous possible types of solvents is given. However, in terms of cost and organization of the gel-technology process itself, two types of solvents are currently used—vaseline (paraffin) oil (Honeywell, Charlotte, NC, USA) and decalin (DSM, the Netherlands). In both cases, approximately the same physical and mechanical characteristics of the product are achieved [25,30]. The specifics of these two processes are considered in [28,30,31,32]. From an economic point of view, it is desirable to use solutions with the highest PE concentration and the highest possible molecular weight. However, with the simultaneous combination of these indicators, it becomes impossible to extrude solutions through real spinnerets due to the very high viscosity of the spinning solutions. Therefore, reactor powders with Mw = (2–3) × 10^6^ are currently used in real technological processes. Usually, the concentration of solutions does not exceed 5–10%.

A great contribution to understanding the nature of the association of macromolecules in dilute UHMWPE solutions and gels was made by J. Delmas [33,34,35,36,37], who studied the dissolution of UHMWPE reactor powders using a “slow” DSC—a very sensitive and stable calorimeter (Setaram, Lyon, France), in which it was possible to place a large cuvette, stir it, and heat it at a very low rate (1–3 K/h). As mentioned above, it was assumed that the stable network knots induced by shear and responsible for the “continuity” of the gel are of a crystalline nature, however, no melting endotherms at high temperatures were previously found on thermograms. Such a slow heating, which could be carried out in Setaram, made it possible to immediately reveal 3 types of crystalline formations in UHMWPE reactor powders: fraction I consists of crystallites with a lower melting point, fraction II (141 °C) refers to unstrained crystallites, and fraction III (above 155 °C) to stressed crystallites. The equilibrium temperature of the end of dissolution depends on the solvent and thermal history and can even reach 165 °C. The high-temperature exotherm obtained upon cooling (160 °C) indicates that a complete randomization of macromolecules does not occur upon dissolution.

The stability of fraction III in the presence of the solvent should affect the properties of the dilute solution, wet gel and xerogel. This fraction is similar to a net, the knots of which are unmelted crystallites stabilized by stress. The percentage of such network can reach up to 25% and depends on many parameters, such as thermal history, M_w_, etc. The existence of temperature-resistant crystals provides a simple explanation for the long-term memory effect of gels and makes one doubt on the key role of entanglements in gel-formation.

The authors of [38] proposed a model in which the drawing ratio was determined not only by the entanglement density, molecular weight, and molecular weight distribution of UHMWPE, but also by the temperature and stretching rate [38,39,40].

In later works [40], the influence of the concentration of the Hifax 1900 solution with M_w_ = 4×10^6^ g/mol on the structure of disordered regions was studied. Using the ^1^H NMR method, it was found that the mobility of amorphous chains was more limited in samples obtained from solutions with a lower concentration, despite the fact that the concentration did not affect the degree of crystallinity and the relaxation characteristics of the crystalline regions. This allowed the authors to suggest that, in such samples, the lamellae are connected by taut-tie molecules. It should be noted that not only the arrangement of amorphous regions (which is very important in itself), but also the perfection of crystalline regions (regularity of folding in the crystallites) as well as the relative arrangement of lamellas play a very important role for the subsequent orientational drawing of the gels.

Usually, orientation drawing (orientation strengthening) of the freshly spun fibers/films is realized in the next stage of the process. On the basis of structural studies, it is shown that the process of orientational drawing consists in a radical rearrangement of the initial isotropic lamellar structure into a completely different, macro- and microfibrillar structure. As a rule, to obtain the fibers with ultimate mechanical properties, the drawing process is carried out in several stages. Therefore, one of the main problems is the scientifically based choice of temperature and force regimes at each stage of orientational hardening. Solving these questions is one of the most important goals of this work.

## 2. Materials and Methods

### 2.1. Samples Preparation

A reactor powders of UHMWPE were synthesized at Boreskov Institute of Catalysis of the Siberian Branch of the RAS (Novosibirsk, Russia) on supported titanium-magnesium catalysts. An average viscosity molecular weight was M_w_ = 3×10^6^ g/mol.

A weighed portion of UHMWPE powder (based on 1.5% solution concentration) together with an antioxidant (0.1 wt.% di-tert-butyl-p-cresol) was placed in a test tube with a solvent and immersed in a thermostat liquid, the temperature of which was raised from 100 °C to the dissolution temperature UHMWPE (120 °C, in the case of decalin and 140 °C, in the case of vaseline oil) with constant stirring. Stirring was stopped as soon as the viscosity of the solution began to increase sharply. Then the stirrer was removed from the solution, and a reflux condenser with a ground stopper was inserted into the test tube, the solution was heated to 160 °C and kept at this temperature for an hour to homogenize the solution. Then the transparent homogeneous solution was poured into a Petri dish at room temperature (or into a Petri dish heated to 100 °C). Due to cooling and subsequent crystallization, the solution passes into a liquid gel state with the formation of a porous structure from randomly oriented interconnected stacks of lamellas (such as a spongy structure).

After drying, the wet gels turn into so-called xerogels, opaque thin films 80–100 µm thick. Xerogels from decalin (xerogel-D) were obtained by removing the solvent by simple evaporation at room temperature for 2–3 days. To remove vaseline oil (xerogel-VO), a more complicated procedure was required: first, the oil was squeezed out under slight pressure between layers of filter paper, and then it was washed several times in gasoline and hexane at *T_room_*, changing the solvent 5–6 times. Next, the xerogel films were cut into narrow strips about 1 mm wide and subjected to multistage high-temperature orientation drawing on a special laboratory setup. By changing the orienting force and increasing the temperature, oriented film threads were obtained with different draw ratios (*λ*) from 10–12 to 100–120 times. The limiting drawing temperature was 139–141 °C, i.e., it reached *T_melt_* of polyethylene in the unloaded state. It is the use of the multistage method of high-temperature zone drawing that makes it possible to obtain ultra-oriented high-strength UHMWPE film filaments.

### 2.2. Scanning Electron Microscopy (SEM)

The structure of the HDPE-based composite fibers has been investigated using the scanning electron microscopes SUPRA 55VP (Carl Zeiss, Oberkochen, Germany). Specimens have been sputtered with a thin Pt layer, placed into the SEM microscope and observed in secondary electrons (SE) mode at accelerating voltage in the range of 5–10 kV.

### 2.3. Wide-Angle X-ray Scattering (WAXS)

The crystalline structure of the UHMWPE samples has been investigated by 2D-WAXS using the diffractometer Bruker D8 DISCOVER (Bruker, Karlsruhe, Germany) with point focus, 0.5 mm spot size and parallel beam filtered CuKα radiation, as well as an Imagine Plate area detector (Anton Paar, Graz, Austria).

### 2.4. Differential Scanning Calorimetry (DSC)

The thermodynamic characteristics of UHMWPE nascent powder samples were studied on a DSC-2 Perkin-Elmer calorimeter with varying heating rates V from 0.3 to 10 K/min. The temperature scale was calibrated by the melting points of ice (273.1 K) and indium (429.7 K), the heat flux scale was calibrated by the heat capacity of sapphire, and the value of the thermal effect, determined from the area on the DSC curve, by the enthalpy of melting of indium (ΔH_f_ = 6.80 cal/g). For calculation of the degree of crystallinity of the prepared samples, the value of melting enthalpy (ΔH^0^_m_) as 294 J/g [41] has been used, which had been earlier found for HDPE with the crystallinity degree of 100%.

### 2.5. Mechanical Testing

Short-term mechanical tests have been carried out at room temperature using a universal tensile machine INSTRON 5943 (Instron, High Wycombe, UK) using special clips according to the ISO 527 Standard at the tensile rate of 10 mm/min. The fibers basic length has been equal to 100 mm. Totally, 10 samples for each fiber type have been examined. Tensile strength (*σ*_br_, GPa) and the strain at break (*ε*_br_, %) have been measured from the obtained stress-strain curves. The measurement error did not exceed 15%.

## 3. Results and Discussion

### 3.1. Study of the Structure of UHMWPE Nascent Powder

The ability of UHMWPE reactor powder to form a good spinning solution from which fibers can be spun depends surprisingly on the structure of the nascent particles, which would have to disintegrate when dissolved in a solvent, since each individual molecule in a dilute solution tends to take the form of a random coil. However, experience shows that there is a so-called “memory” of the nascent polymer, and not all reactor powders can be used to obtain a good spinning solution and form fibers that, after orientation hardening, will demonstrate high mechanical characteristics. Even if the reactor powders have similar molecular weights and molecular weight distributions, then some powders cannot be spun, others are spun, but the fibers obtained from them do not orient well, and only from individual reactor powders it is possible to obtain final fibers with high physical and mechanical characteristics. Electron microscopy studies make it possible to study the fine structure of the nascent particles of the reactor powders and compare it with the nature of powder dissolution in a solvent and gel-formation.

**Figure 1:**
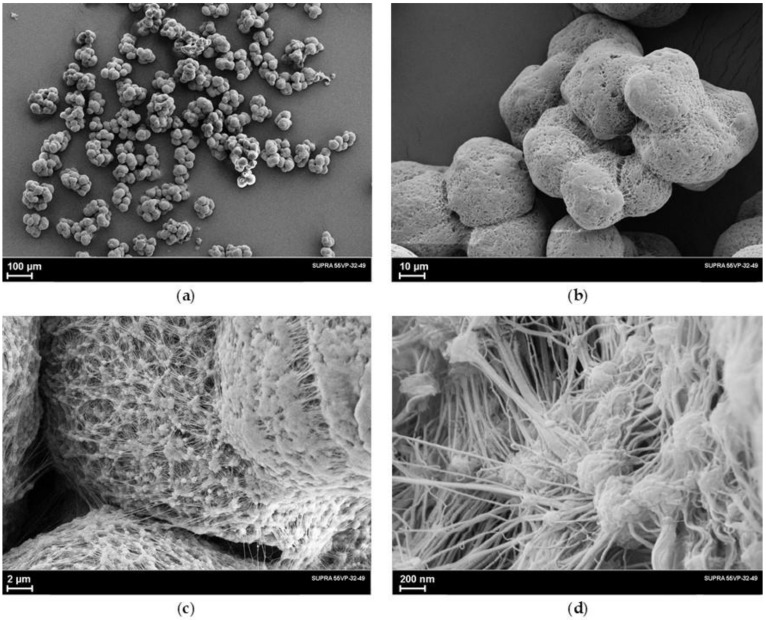
SEM micrographs of the UHMWPE reactor powder taken at different magnifications (**a**–**d**).

Figure 1a–d shows SEM images at various magnifications. The analysis of these images shows that the nascent particles of the studied reactor powder have an irregular shape and are a conglomerate consisting of smaller, approximately spherical particles. SEM images at high magnifications (Figure 1c,d) show that the individual particles of the reactor powder are actually an extremely complex system of interconnected smaller particles due to numerous microfibrils with a diameter of about 6–50 nm. It can also be seen that the fibrils not only extend from the boundary of one small particle to another, but they form a 3-dimensional network owing to the presence of very small spherical formations with a diameter of 20–50 nm, which play the role of a kind of net nodes.

It is of great importance to elucidate of the nature of the appearance of fibrillar formations in the reactor powders. As is known, two processes occur during synthesis—an increase in the length of a macromolecule at the active center of a catalyst particle and subsequent crystallization of the formed macromolecules. Until now, there is no unified view of how the crystallization of many molecules occurs, which grow simultaneously on numerous active centers of the same catalyst particle. Based on our SEM images of the reactor powder and taking into account the data on the size of the catalyst particles, which are only a few microns, we proceed from the following scheme for the formation of a multilevel hierarchical structure of the reactor powder particles:

- catalyst particles with a diameter of several microns form in the polymerization medium some associates of several tens of particles, which further determines the size of the already formed polymer particles;
- on each catalyst particle this agglomerate, the process of synthesis and crystallization of UHMWPE macromolecules simultaneously begins. The presented above SEM microphotographs, taken at different magnifications, allow us to assume that during crystallization, lamellae of the folded macromolecules layering on top of each other are formed;
- since the location of the catalyst particles in the agglomerate is fairly close, the growing macromolecules and lamellae interpenetrate each other, so that a complex structure of the reactor powder, interconnected in three directions, is formed;
- due to different rates of polymerization and crystallization processes on the individual catalyst particles and a non-uniform increase in the volume of the formed microspheres (or the occurrence of local temperature gradients inside the conglomerate because of heat release during synthesis), the entire set of interconnected polymer microspheres is “bloated”. It should inevitably lead to stretching of the folded lamellae leading to the formation of microfibrils. Moreover, the lamellas are primary structural elements, and the microfibrils are secondary structures.

This structure provides a large porosity of the particle and facilitates the rapid and simultaneous penetration of the solvent to the elementary structural units, which, apparently, determines the good properties of the spinning solution from the UHMWPE powder studied in this work.

It should be noted that there is also a significant amount of fibrillar formations in the morphology of UHMWPE reactor powders used in the production of high-strength and high-modulus fibers by the gel-technology method by commercial firms DSM, the Netherlands (Stamylan powder), and Honeywell, USA (MitSui powder).

**Figure 2:**
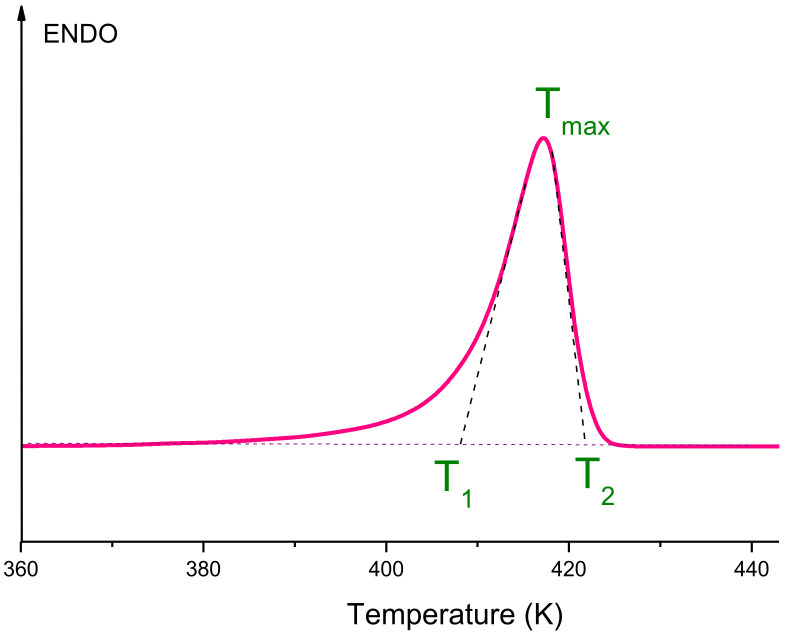
An example of the melting thermogram of the UHMWPE nascent powder.

Additional information about the structure of the UHMWPE reactor powder was obtained using the DSC method. All melting curves obtained at various heating rates (V) were carefully processed and analyzed. One of them is presented, as an example, in Figure 2.

**Figure 3:**
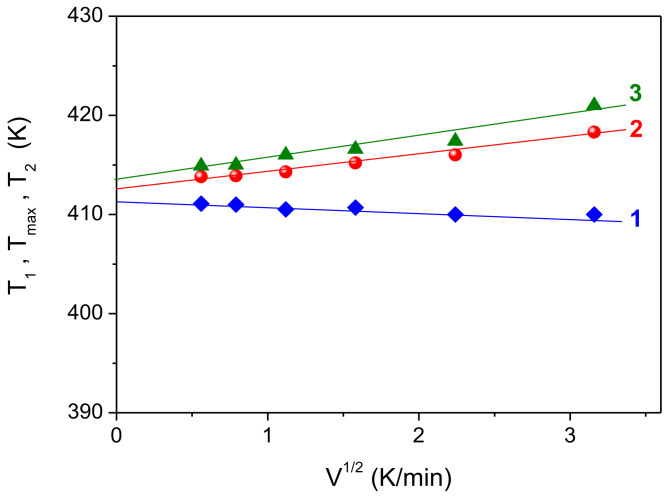
Dependences of temperature parameters *T*_1_ (**1**), *T_max_* (**2**) and *T*_2_ (**3**) of the melting peak of UHMWPE nascent powder samples of the same mass (6 mg) on the heating rate *V*^1/2^.

To determine the true temperature parameters of the melting peak (the beginning of the melting peak *T*_1_, the temperature of the maximum of the melting peak *T_max_*, the temperature of the end of the melting peak *T*_2_) from DSC curves, it is necessary to plot the dependences of these parameters on the heating rate (*V*), namely *T*_1_(*V*^1/2^), *T_max_*(*V*^1/2^), *T*_2_(*V*^1/2^). This procedure allows one to avoid effects caused by thermal lag at different sample heating rates. If these dependences are linear, it is necessary to extrapolate them to zero heating rate. Such dependences are shown in Figure 3. It can be seen that the experimental points for the dependences *T*_1_(*V*^1/2^), *T_max_*(*V*^1/2^) and *T*_2_(*V*^1/2^) fit fairly well on the straight lines (1–3), and their extrapolation to zero heating rate gives the true temperatures *T*_1_ = 411.6 K, *T_max_* = 412.6 K and *T*_2_ = 413.6 K. Note that the slope of the straight lines in this case is determined by the mass of the sample, which remained constant in our experiment.

Thus, based on the studies of the thermodynamic characteristics of the UHMWPE reactor powder, it can be concluded that this powder has a high degree of crystallinity (70%), and its true melting point is significantly higher than the melting point of UHMWPE crystallized from the melt (usually about 408–409 K), which indicates the presence of a large number of straightened segments of the macromolecules.

### 3.2. Study of the Structure of UHMWPE Xerogels

**Figure 4:**
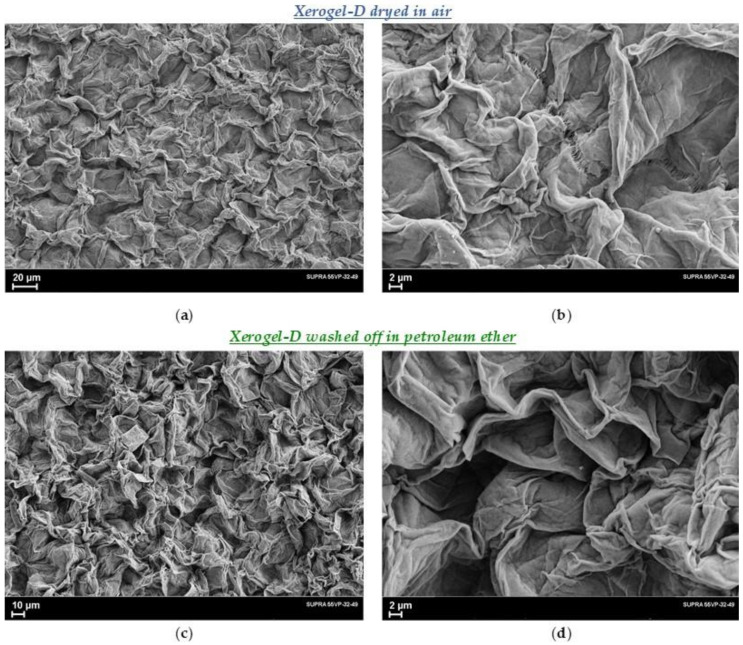
SEM micrograph of the xerogel-D films obtained from a 1.5% solution in decalin: (**a**,**b**) dried at *T_room_* in air for a week; (**c**,**d**) washed from the solvent in petroleum ether at *T_room_*.

A comparative study of the morphology of the xerogels obtained from 1.5% solutions in decalin and vaseline oil was carried out. Figure 4 shows SEM micrographs of the xerogel-D obtained from the 1.5% solution in decalin. Decalin was removed from the samples by two ways: (1) evaporation at *T_room_* for a week; (2) washing off in petroleum ether at *T_room_*. The micrographs taken at different magnifications clearly show the typical structure of films dried from gels, repeatedly observed by different authors [3,12,26] in similar samples. On the surface of the xerogels-D, there are arranged polymer petals, which, apparently, are stacks of lamellar formations, as follows from X-ray diffraction and transmission electron microscope studies [31,33] of similar samples. These studies show that the entire volume of the xerogel film consists of lamellae layered on top of each other, oriented with their basal planes parallel to the plane of the film.

As follows from the comparison of the presented micrographs, the drying process did not change either the appearance or the dimensions of the structural elements. This is not surprising, because the structure formed during polymer crystallization depends primarily on the crystallization temperature and cooling rate. One could expect only some change in the porosity of the xerogels-D, but it is not possible to obtain reliable results in this regard on the basis of electron microscope images alone.

**Figure 5:**
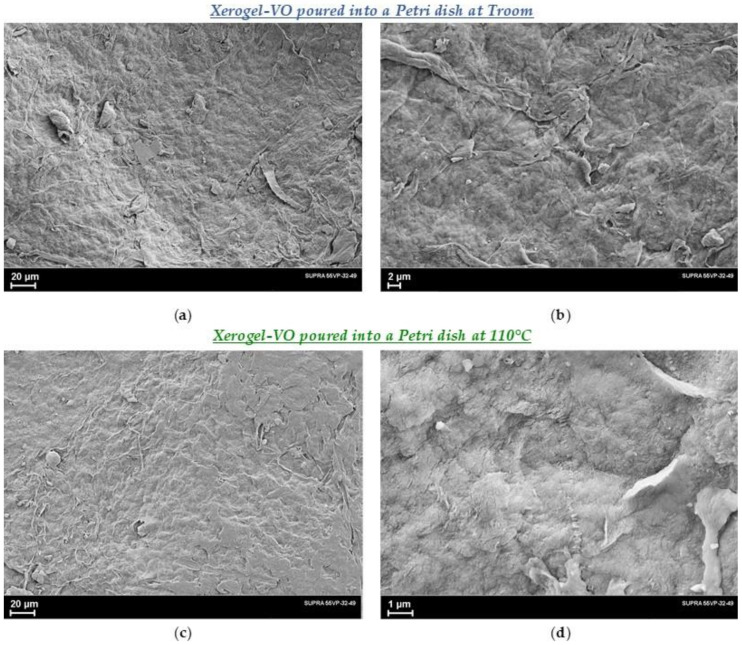
SEM images of the xerogel-VO obtained from a 1.5% solution in vaseline oil: (**a**,**b**)—poured into a Petri dish at *T_room_*, or (**c**,**d**)—poured into a Petri dish at 110 °C, then cooled to T_room_, removed excess oil under pressure, and after that washed off the oil in hexane.

Unlike decalin, vaselin oil cannot be removed from the gel by simple evaporation. Moreover, it cannot be completely removed from the xerogel by repeated washing in gasoline or any other solvent. Figure 5a,b shows micrographs of the xerogel-VO obtained from a 1.5% solution in vaseline oil when pouring the solution into a Petri dish at room temperature. The excess oil was first squeezed out of the gel, and then it was washed in hexane at *T_room_* and dried between filters under a small weight. Figure 5c,d shows SEM images of the xerogel-VO obtained in the same way, with the only difference that the solution was poured into a Petri dish heated to a temperature of 110 °C, which then cooled down to *T_room_*.

Compared to xerogel-D films, the structure of these xerogel-VO samples is indistinct and it is difficult to reveal any differences between xerogels obtained with different cooling rates. It can only be said that the films prepared using vaseline oil as a solvent have a denser (ie less porous) structure after drying procedure.

**Figure 6:**
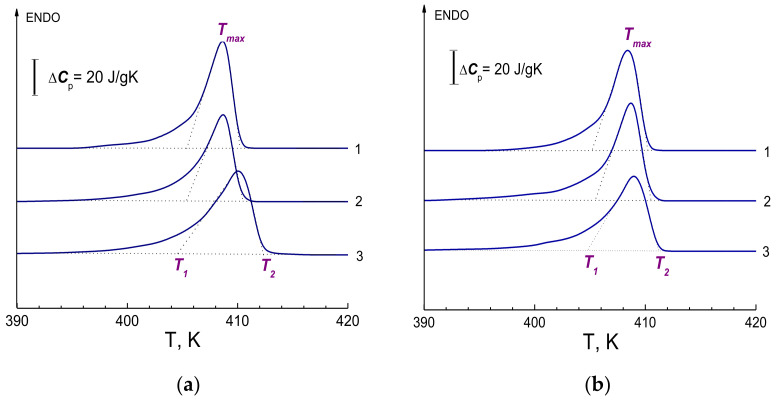
DSC curves obtained on the xerogel samples: (**a**) from decalin; (**b**) from vaseline oil. The data were obtained for samples of the same weight (2.5 mg) at different heating rates: 0.31 K/min (curve 1); 1.25 K/min (2); 5 K/min (3).

Figure 6a,b show a series of several DSC curves obtained on xerogel samples prepared from 1.5% solutions using decalin and vaseline oil. To determine the true temperature parameters of the melting peak, the dependences *T*_1_(*V*^1/2^), *T_max_*(*V*^1/2^), and *T*_2_(*V*^1/2^) were also plotted from these curves. As mentioned earlier, such procedure makes it possible to avoid effects caused by thermal lag at different sample heating rates. The presence of such a thermal delay can manifest itself, in particular, in the shift of the melting peaks towards higher temperatures at increased heating rates *V*—as a result, the melting temperature in curves 3 obtained at a higher heating rate turns out to be higher than in curves 1. Calculation of the melting enthalpy of xerogel samples prepared with decalin and vaseline oil gives the values Δ*H* = 218 J/g and Δ*H* = 219 J/g, respectively. The degree of crystallinity determined from the obtained value of the melting enthalpy was *χ* = 75% in both cases. The parameters of true temperatures *T*_1_, *T_max_*, *T*_2_, melting enthalpy Δ*H* and degree of crystallinity *χ* for xerogel samples are given in Table 1.

A comparison of the data in Table 1 for the nascent powder and xerogels demonstrates an apparent contradiction—the melting point of the nascent powder is higher, while the degree of crystallinity (*χ*) is lower than that of the xerogels. This apparent contradiction is explained by the difference in certain morphologies. In the reactor powder, a significant fraction of the volume is occupied by fibrils while in the both xerogels, lamellar formations predominate. In fibrillar structures, the melting of the lamellae is hampered by a relatively large number of passing macromolecules, which bind the lamellae together and prevent their transition to a disordered state during melting. It was shown in [42,43] for ultimately drawn oriented UHMWPE fibers that such an obstacle leads to an increase in *T_max_*. The difference in the degree of crystallinity is explained by different conditions for the formation of morphologies. During synthesizing the powder in a reactor, the conditions for the formation of morphology are more nonequilibrium than during preparing the xerogels. DSC data were used to evaluate another characteristic of the structure of polymer—the parameter of intrachain melting cooperativity (*ν*), the physical meaning of which is that it determines the minimum sequence of *ν* repeating units in the chain, passing as a whole from a crystallite to a statistical coil in melt [44,45]. The estimations of *ν* was made according to the formula

*Ν* = 2*R* (*Τ_max_*)^2^/Δ*Τ*·Δ*H_f_*^0^,

where *R* is the gas constant. The dimensionless parameter *ν* is expressed by the number of CH_2_ groups in the trans-region of the UHMWPE chain, simultaneously participating in the melting event. If one multiplies *ν* by the length of a single C-C bond (h = 0.124 nm), then this parameter (*L^calc^* = *ν·h*) can be compared with the thickness of lamellar crystals. For the reactor powder and both xerogels, the thickness of lamellar crystals determined from the obtained values of the cooperativity parameter *ν* is *L^calc^* = 15–22 nm (see Table 1). From the data obtained using the DSC method, it can be concluded that xerogels-D are more perfect than xerogels-VO. Despite the fact that they have the same degree of crystallinity (*χ* = 75%), the xerogel-D has a higher melting point *T_max_*, the melting cooperativity parameter *ν* is higher, and the half-width of the melting peak is narrower than that of the xerogel-VO.

### 3.3. Study of Oriented UHMWPE Films Obtained by Gel-Technology

It has been established earlier [6] that, during high-temperature orientational drawing, a radical rearrangement of the initial isotropic nonoriented structure (lamellar) into a new anisotropic macro- and microfibrillar organization occurs. Fibrils appear at the initial stages of drawing, in the “neck” region, as a result of a solid-phase transition: folded chain crystals ⇒ extended chain crystals. Phenomenologically, the transition from an unoriented state to an oriented state looks like a process of “neck” formation, i.e., a sharp abrupt local contraction of the sample, in which the orientation value and the degree of stretching are much higher than those of the rest of the polymer. After the formation of the neck, further orientation of the sample proceeds by extending the neck over the entire length of the polymer. But even after this, further elongation of the sample by means of plastic deformation is possible. Molecular axes, i.e., ***c***-axis of the crystal lattice, acquire a preferential orientation in the drawing direction. Usually, the drawing ratios in the neck region are small (about *λ* = 7 ÷ 10 ÷ 15 times), depending on the type of the lamellar structure formed when the gel-state occurs. With further drawing of the samples, structural changes are of a completely different nature, namely, plastic deformation (slip) of the formed macro- and microfibrils occurs relative to each other. The limiting drawing ratios can reach very large values, up to many tens—several hundreds of times. In this work, values of *λ* ≈ 100–120 times were achieved.

**Figure 7:**
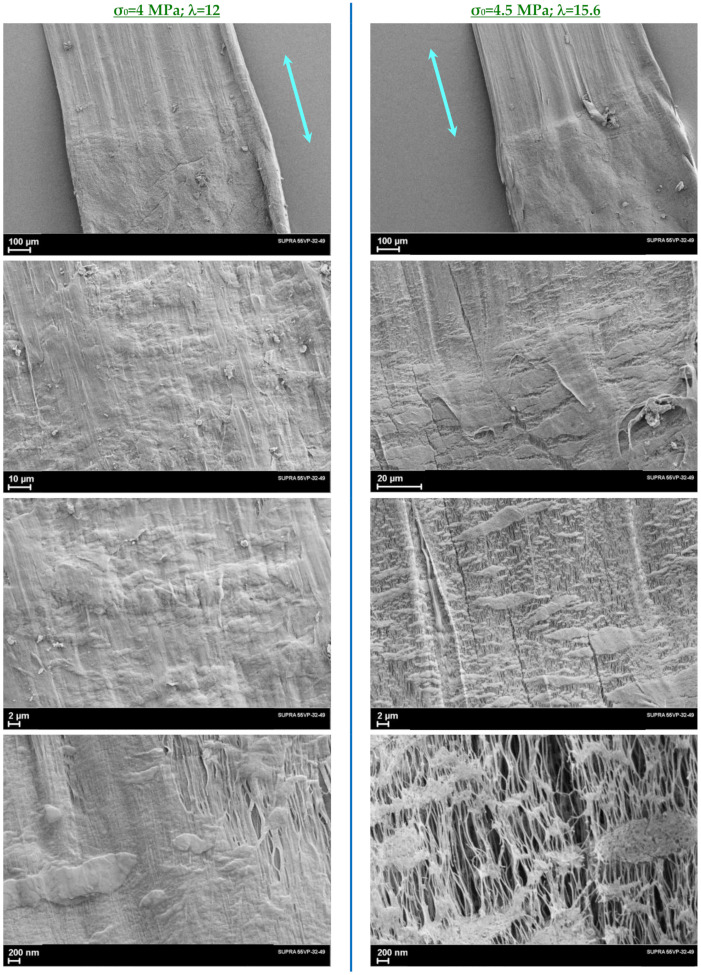
SEM images of the necking region in the UHMWPE films recorded at different magnifications: **left side**—*λ* = 12, initial applied stress *σ_0_* = 4 MPa; **right side**—*λ* = 15.6, *σ*_0_ = 4.5 MPa. Blue arrows indicate drawing direction of the films.

Figure 7 shows the obtained SEM images of the transformation of the initial structure of the gel-cast UHMWPE films into the microfibrillar structure of the oriented state. In the neck region, a sharp transition from one type of structure to another one is clearly visible. At the same time, a significant difference is seen in the course of the structure rearrangement process depending on the initial applied stress (*σ*_0_): the higher *σ*_0_, the more intense the transition of the lamellar structure to the microfibrillar one. Although it is obvious that in both cases, some islands with non-oriented lamellar morphology remain in the neck, which will turn into microfibrils during the subsequent drawing stages. The microfibrils are the main elementary morphological formations in the oriented fibers and films. Their transverse dimensions are 10–20 nm; the cross section contains about 400–500 macromolecules, and the lengths of microfibrils reach values of at least several micrometers. It is also well known [6] that crystalline and disordered regions alternate along the long axis of the microfibrils. It is the structure of the disordered parts that controls the mechanical strength of the oriented polymer. During the transition from non-oriented polymer to oriented polymer, in the neck region, the microfibrils are already mostly oriented along the drawing direction, i.e., along the fiber axis.

**Figure 8:**
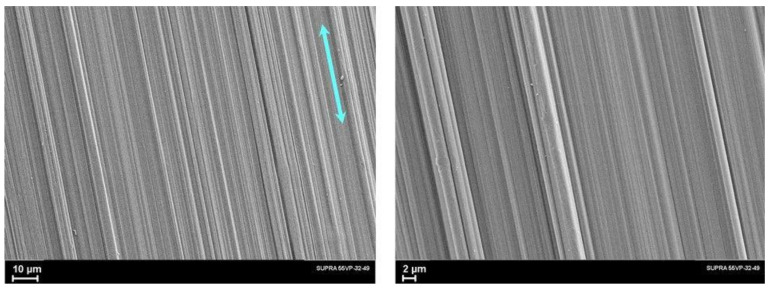
SEM images of the oriented UHMWPE film (*λ* = 77) made at different magnifications. The blue arrow shows the drawing direction.

At the second stage of orientational drawing, plastic deformation of the microfibrillar structure occurs due to the slippage of the fibrils along each other. In this case, along with tensile forces, transverse compressive forces act, which compact the fibrillar structure and ensure the development of shear forces inside the fibrils. Figure 8 shows micrographs of the oriented UHMWPE film with a draw ratio of *λ* = 77. The images at high magnification clearly show anisodiametric formations oriented along the film orientation axis. Their transverse size is larger than the diameter of the microfibrils observed in the “neck” micrographs (Figure 7). These are the so-called macrofibrils, i.e., bundles of microfibrils, which were formed from one stack of lamellae. Under the action of shear forces developing in the process of fibrils’ slippage, the structure of disordered intrafibrillar regions changes. This fact, as well as a decrease in the misorientation of the crystallites relative to the drawing axis, leads to improving the mechanical properties.

**Figure 9:**
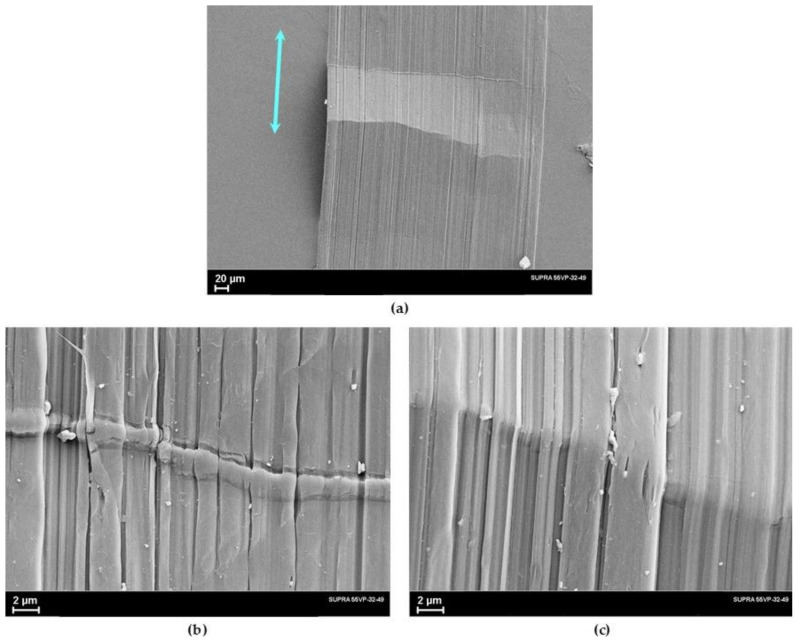
SEM images of the kink-bands on oriented UHMWPE film: (**a**) low magnification; (**b**,**c**) high magnification.

It is also known [6] that during the orientational stretching of a polymer under non-optimal conditions, large rotational defects (so-called kink-bands) can extend across the film orientation axis. They are capable of leading to sample failure long before the limiting drawing ratios are reached. The appearance of the kink-bands is preceded by ruptures of the individual micro- and macrofibrils. Examples of such kink-bands are presented in Figure 9.

This phenomenon was studied in detail in the works of M.F.Milagin. When a separate fibril breaks, the entropy energy stored in it is released. Unloading waves propagate through the sample up to the fixed clamp, reflect from it and interact with the direct wave, which leads to the generating of compressive forces. Compression of long rods (i.e., the microfibrils) in the direction of their long axis leads, as is known, to loss of Euler’s stability. If the structure of the microfibrils were perfect, then under the action of the compression forces, the microfibrils would be completely destroyed. However, the microfibrils have disordered areas, which give them plasticity. Owing to the presence of a significant number of the defective areas, sharp fractures of the microfibrils become possible. One can also expect delamination of thin layers of oriented material, which is confirmed by direct SEM observations.

Theoretical analysis [46] has shown that the propagation of the kink-bands in the direction transverse to the orientation axis is due to the instability of the plane front of the kink-band: there is a region in front of the peak, in which the front stresses are sufficient to initiate kink-bands formation in neighboring regions of the oriented polymer. The kink-bands slowdown in the material and, at the same time, they end having a wedge-shaped front with an extremely small narrowing angle [6].

It has been found elsewhere [47] that ultimately oriented polymers can be streaked with transverse and longitudinal microcracks as a result of the kink-bands formation. Even single bends at small angles of such samples lead to intensive development of the kink-bands formation.

Using the SEM method, it was found [1,4] that in the places of a sharp fracture of individual microfibrils at the boundaries of the kink-bands, localized occurrence of closely spaced submicrocracks 200–300 A in size occurs; original foci are created with a dangerous local concentration of submicrocracks, which combine into pointed microscopic cracks from 0.5 µm to several µm in length. Ultimately, a main crack appears, leading to the failure of the sample. The average distance between the kink-bands can reach 1–3 µm. They have a large extent in the transverse direction (up to tens of micrometers).

### 3.4. Analysis of the Thermodynamic Characteristics of the Oriented UHMWPE Films by DSC

**Figure 10:**
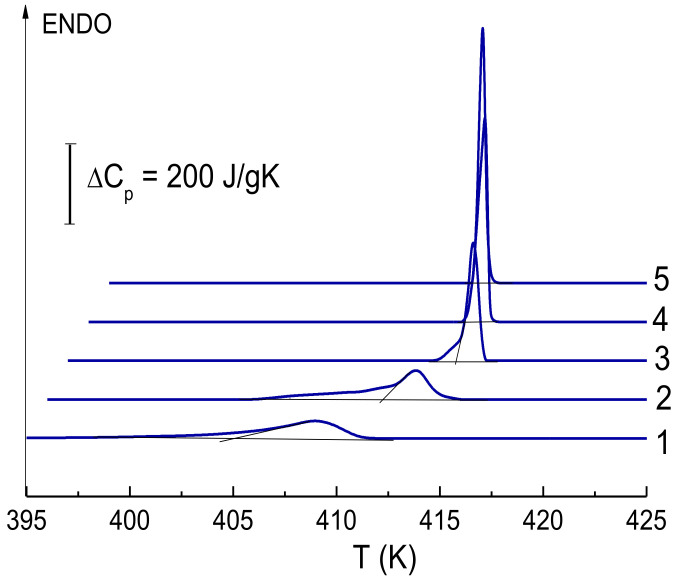
DSC curves obtained at a heating rate of V = 5 K/min for samples: 1—initial xerogel film (vaseline oil); 2—*λ* = 9; 3—*λ* = 43; 4—*λ* = 95; 5—*λ* = 170.

Figure 10 shows, for example, DSC curves for one of the studied samples, which clearly show the nature of the change in the thermodynamic parameters of the melting peak during drawing.

Thus, the temperatures *T*_1_, *T_max_* and *T*_2_ increase, and the shape of the peak changes in the following manner: the amplitude grows and at the first stage (*λ* < 50) a low-temperature shoulder appears—all these is associated with the unfreezing of mobility in the extended parts of the molecules that connect the crystallites to each other. At high degrees of drawing, the shoulder disappears because of the transition of these molecule parts from the amorphous state to the crystalline one. The quantitative characteristics of the change in the thermodynamic parameters of the melting peak were determined in a series of experiments to determine the experimental dependences *T*_1_(*V*^1/2^), *T_max_*(*V*^1/2^), *T*_2_(*V*^1/2^) and their extrapolation to zero heating rate *V*. Data on the true temperature parameters of the melting peak, melting temperature range and melting enthalpy are given in Table 2. The calculated values of the intrachain melting cooperativity parameter (*ν*) and the thickness of lamellar crystals (*L^calc^*) are also given there.

As it can be seen from the data given in Table 2, the most conservative parameter is the melting temperature *T_max_*, which increases within 1–2.5 K as the draw ratio enhances. The melting enthalpy Δ*H* and the degree of crystallinity *χ* are found to rise at high degrees of drawing by 10–30%. It should be noted that at relatively low draw ratios starting from the necking draw ratio (*λ* = 9–10) and up to *λ* = 45, these both parameters slightly decrease in comparison with the data on xerogels (see Table 1).

**Figure 11:**
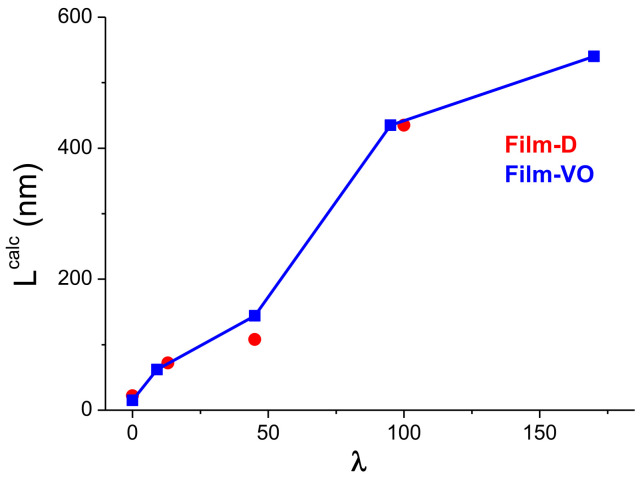
Dependence of the parameter *L^calc^* on the draw ratio of the films from xerogel-D and xerogel-VO.

The most radical changes occur with the melting temperature range Δ*T* = *T*_2_ – *T*_1_ and associated parameters *ν* and *L^calc^*. The data in Table 2 demonstrate that the values of the intrachain cooperativity of melting (*ν*) and crystal sizes (*L*) increase tenfold as the draw ratio increases. At *λ* = 170, the size of the crystalline formations in the films obtained from the xerogel reaches ~0.5 microns. Referring to Figure 11, it is found that the increase in the parameter *L^calc^* for these samples occurs in two stages. At the first stage, a “neck” appears and the polymer structure is formed, which is transitional from lamellar to fibrillar. The latter is characterized by a large number of the taut tie segments of the molecules that connect the crystallites to each other. The thermodynamic state of these segments of molecules is liquid; therefore, they do not make an additional contribution to the enthalpy of fusion of the entire sample. At the second stage (*λ* > 50), these segments of molecules pass from the liquid state to the crystalline one, and, as results, one observes an increase not only the parameter *L^calc^*, but also the melting enthalpy of the entire sample. Considering that there is a direct relationship between the parameters *ν* and *L^calc^* with the strength characteristics of polyethylene [42,43], a significant increase in strength in the second stage (*λ* > 50) for the drawn samples obtained from the both types of the xerogel films can be expected.

### 3.5. Analysis of the Orientation Degree of the Crystallites in UHMWPE Films by WAXS

In the present work, X-ray photographs were analyzed, which were obtained on the drawn UHMWPE films. For polyethylene at room temperature and atmospheric pressure, an orthorhombic cell with parameters is thermally stable: *a =* 7.4 Å, *b*= 4.96 Å, *c* = 2.54 Å, *α* = *β* = *γ* = 90°.

If the crystalline regions in the sample are located randomly relative to each other (isotropic, non-oriented material), the diffraction reflections from each crystallite merge into continuous rings, so-called Debye’s rings. If the crystallites line up along any direction in space under some impacts (for example, a uniaxial orientation drawing), then the ring reflections will transform into anisotropic arcs, the azimuthal width of which can serve as a quantitative characteristic of the degree of crystallite orientation along this direction. For an orthorhombic cell, reflections 100 and 200 are the most intense, and therefore they are most often used to analyze X-ray patterns.

**Figure 12:**
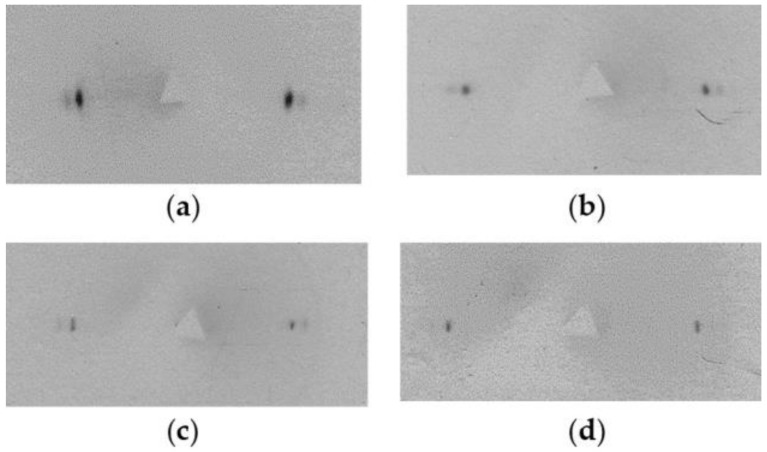
2D-WAXS patterns for the oriented UHMWPE films obtained from a 1.5% solution in decalin with the values of the degree of drawing: (**a**) *λ* = 14; (**b**) *λ* = 63; (**c**) *λ* = 89 and (**d**) *λ* = 119. Orientation of the samples was vertical.

Figure 12 shows the series of X-ray diffraction patterns obtained from samples oriented to different draw ratios. As can be seen from the X-ray diffraction patterns, the crystallites after the transformation of the initial structure into a fibrillar one (which occurs immediately after the appearance of the neck when the draw ratio reaches 14) are oriented along the drawing direction. The misorientation angle of crystallites in the samples is very small and amounts to 2–3°. After the propagation of the neck along the films, further drawing leads to a decrease in the difference in length of molecular segments in the amorphous phase and some increase in the size of crystallites, which is seen from a decrease in the half-width of equatorial reflections.

As can be seen, there are no differences in the angular position of the equatorial reflections for the samples with different draw ratios. Therefore, starting from the necking stage, the orientation of crystallites in the oriented UHMWPE films is very high, and it practically does not change during further drawing up to *λ* = 120.

### 3.6. Results of Mechanical Testing of Oriented UHMWPE Films

**Figure 13:**
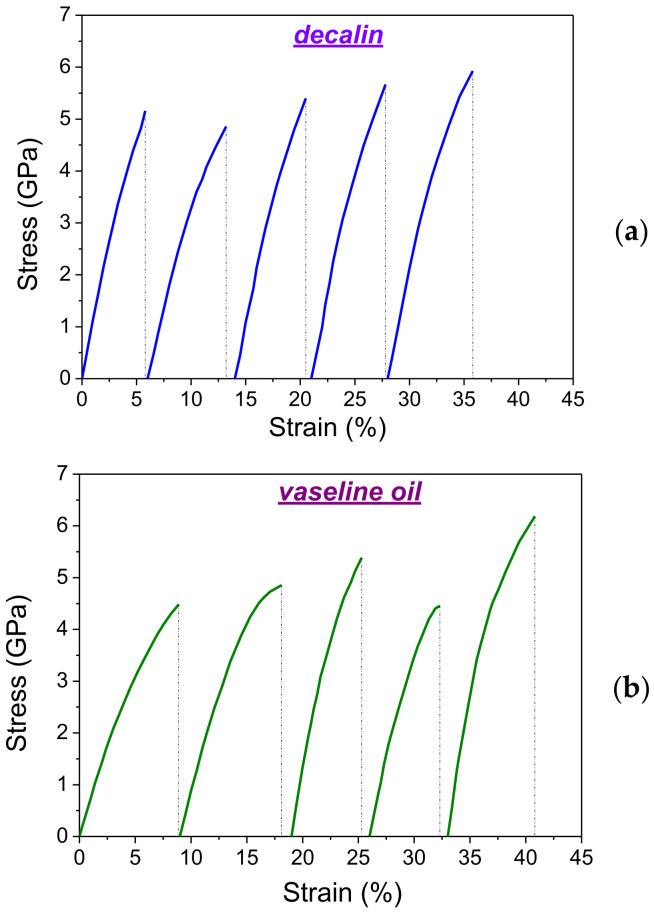
Stretch diagrams for 5 samples of highly oriented UHMWPE films obtained from gels in decalin (**a**) and vaseline oil (**b**). For clarity, the curves are shifted relative to each other along the *x*-axis.

Typical stress-strain curves for the highly oriented UHMWPE films formed from 1.5% gels in decalin and 1.5% vaseline oil are shown in Figure 13. It can be seen that all curves have the same and well reproducible shape, and their shape does not depend on the type of solvent used. When processing the tensile curves, the strength (*σ_br_*) and deformation at break (*ε_br_*) were determined, as well as Young’s modulus (*E*).

The values of *σ_br_*, *ε_br_* and *E* were determined from the results of measurements on 50 samples with different draw ratios for 2 types of solvents used (see Table 3). The mechanical characteristics of ultimately drawn UHMWPE films are found to differ little from each other (within the error) both when decalin and vaseline oil are used as solvents. However, it is worth noting that a number of samples (about 6%) showed superhigh values of tensile strength (5.8–6 GPa) and Young’s modulus (up to 200 GPa).

## 4. Conclusions

For the highly oriented UHMWPE films obtained using decalin and vaseline oil as the solvents, the values of the tensile strength and Young’s modulus were 4.7–5 GPa and 170–180 GPa, respectively. For some studied samples, the strength values reached 5.8–6.0 GPa, and the Young’s modulus—up to 200 GPa. The achieved values of strength and modulus are among the highest according to the world scientific and patent literature.

The physical basis of obtaining high-strength and high-modulus UHMWPE films is the kinetic concept of the mechanism of destruction of solids, the nature of structural transformations occurring in polymers under the influence of load and temperature, and the concept of the important role of the supramolecular structure of polymers in the process of orientational hardening.

## Figures and Tables

**Table 1:** Thermodynamic parameters of the melting peak of the samples of UHMWPE nascent powder and two xerogels (in decalin and vaseline oil).

Sample	*T*_1_, K	*T_max_*, K	*T*_2_, K	Δ*Τ*, K	*ν*	*L^calc^*, nm	Δ*H*, J/g	*χ,* %
Nascent powder	411.6	412.6	413.6	2.0	175	21	206	70
Xerogel-D	407.0	408.2	408.9	1.9	180	22	218	75
Xerogel-VO	405.6	407.5	408.4	2.8	120	15	219	75

**Table 2:** Thermodynamic parameters of the melting peak of the samples from xerogels with different drawing ratios *λ*.

*λ*	*T*_1_, K	*T_max_*, K	*T*_2_, K	Δ*Τ*, K	*ν*	*L^calc^*, nm	Δ*H*, J/g	*χ*, %
1 wt.% in vaseline oil								
9	412.3	412.7	413.0	0.7	500	62	202	69
45	415.0	415.2	415.3	0.3	1170	145	205	70
95	415.55	415.5	415.45	0.1	3500	435	256	82
170	415.25	415.20	415.17	0.08	4400	540	262	90
1.5 wt.% in decalin								
13	412.9	413.2	413.5	0.6	580	72	220	76
45	413.9	414.1	414.3	0.4	870	108	220	76
100	415.05	415.1	415.15	0.1	3500	435	255	87

**Table 3:** Mechanical properties of oriented UHMWPE films.

	*σ_br_*, GPa	*ε_br_*, %	*E*, GPa
Decalin samples	4.6 ± 0.4	3.1 ± 0.4	182 ± 17
Vaseline oil samples	5.0 ± 0.7	3.2 ± 0.5	170 ± 15

## Data Availability

Not applicable.
